# Efficacy and Safety of Zonisamide in Dementia with Lewy Bodies Patients with Parkinsonism: A Post Hoc Analysis of Two Randomized, Double-Blind, Placebo-Controlled Trials

**DOI:** 10.3233/JAD-200893

**Published:** 2021-01-19

**Authors:** Kazuko Hasegawa, Kenji Kochi, Hidenori Maruyama, Osamu Konishi, Shunji Toya, Toshinari Odawara

**Affiliations:** a Neurology, National Hospital Organization, Sagamihara National Hospital, Kanagawa, Japan; b Data Science, Sumitomo Dainippon Pharma Co., Ltd., Tokyo, Japan; c Medical Affairs, Sumitomo Dainippon Pharma Co., Ltd., Tokyo, Japan; d Health Management Center, Yokohama City University, Kanagawa, Japan

**Keywords:** Dementia, lewy bodies, parkinsonism, zonisamide

## Abstract

**Background:**

Although previous phase II and III clinical trials conducted in Japan showed that zonisamide improved parkinsonism in patients with dementia with Lewy bodies (DLB), some differences in efficacy outcomes were observed between the trials.

**Objective:**

We aimed to further examine the efficacy and safety of zonisamide in DLB patients with parkinsonism in a *post hoc* analysis of pooled data from the previous phase II and III trials.

**Methods:**

Both trials featured a 4-week run-in period followed by a 12-week treatment period with a double-blind, placebo-controlled, parallel-group, randomized, multicenter trial design. In our pooled analysis, the primary outcome was the change in Unified Parkinson’s Disease Rating Scale (UPDRS) part III total score. Other outcomes included the changes in Mini-Mental State Examination (MMSE) and Neuropsychiatric Inventory-10 (NPI-10) scores, and the incidence of adverse events.

**Results:**

Zonisamide significantly decreased the UPDRS part III total and individual motor symptom scores but did not affect the MMSE or NPI-10 scores at week 12. There was no difference in the incidence of adverse events between the zonisamide and placebo groups except for decreased appetite, which had an increased frequency in the zonisamide 50 mg group compared with placebo.

**Conclusion:**

Our findings indicate that zonisamide improved parkinsonism with DLB without deterioration of cognitive function and or worsening behavioral and psychological symptoms of dementia.

## INTRODUCTION

Dementia with Lewy bodies (DLB) is the second most common type of dementia in older adults after Alzheimer’s disease, accounting for 10% –15% of all dementia cases [1]. The clinical features of DLB are a progressive cognitive decline accompanied by fluctuating cognitive function, visual hallucinations, parkinsonism, and rapid eye movement sleep beh-avior disorder [2]. A recent revision of recommendations on the clinical diagnosis of DLB delineates the clinical features more clearly from diagnostic biomarkers while providing guidance on how best to establish and interpret these parameters [2].

The pathology of parkinsonism in DLB is similar to that in Parkinson’s disease (PD), with both fea-turing the loss of dopaminergic neurons in the nig-rostriatal pathway and presence of Lewy bodies [3–5]. Major characteristics of parkinsonism in DLB include bradykinesia and rigidity, and less frequently, tremor at rest [3, 5, 6]. Given that these motor symptoms are associated with diminished activities of daily living (ADL), falls, difficulty swallowing, and high financial costs of care [7–12], it is clinically beneficial to improve parkinsonism in patients with DLB. Furthermore, ADL is correlated with quality of life and caregiver burden [11, 13], and improvements in ADL can play an important role in improving overall patient outcomes.

Clinical management of parkinsonism in DLB typically includes levodopa, the standard therapy for PD and commonly administered to patients with DLB. However, patients with DLB appear to be less responsive to levodopa compared with patients with PD, and the risk of exacerbation of psychiatric symptoms remains a dose-limiting factor [3, 14–16]. Current evidence for the management of parkinsonism in DLB is limited [14], and no unified evidence-based management strategy for parkinsonism in DLB has been established to date.

Zonisamide, approved globally for the treatment of epilepsy, has been approved for the treatment of PD in Japan, where it is typically prescribed as adj-unctive therapy to levodopa [17–20]. The proposed pharmacologic mechanisms responsible for the anti-parkinsonian activity of zonisamide include both dopaminergic (activation of dopamine synthesis and release [21] and inhibition of monoamine oxidase-B [22]) and non-dopaminergic (blockade of sodium channels [23] and T-type calcium channels [24]) functions. In addition, neuroprotective effects of zonisamide have been reported [25–27].

The clear similarities between PD and DLB [5] and the results of an initial series of cases [28] have indicated that zonisamide might be effective for the treatment of DLB patients with parkinsonism. In phase II [29] and III [30] clinical trials within this patient population, zonisamide was shown to improve parkinsonism, assessed by Unified Parkinson’s Disease Rating Scale (UPDRS) part III total score, with a clear benefit on motor function compared with placebo. Furthermore, no notable differences in the incidence of psychiatric adverse events (AEs) were observed between the zonisamide and placebo groups. Based on these outcomes, zonisamide was approved for the treatment of DLB patients with parkinsonism in Japan. However, some differences in efficacy outcomes were observed between the pha-se II and III clinical trials. Zonisamide 25 mg did not significantly improve parkinsonism in the phase II trial but improved it significantly in the phase III trial. With respect to cognitive function, zonisamide did not decrease the Mini-Mental State Examination (MMSE) score in the phase II trial, while zonisa-mide 50 mg significantly decreased the MMSE score compared with placebo in the phase III trial. In addition, the efficacy characteristics of zonisamide in the treatment of DLB patients with parkinsonism, such as the responder proportion and the effect on individual motor symptoms, remain unclear. Furthermore, AEs associated with zonisamide that are significantly more frequent than those observed with placebo are unknown. The differences observed between the above two trials may be attributable to insufficient statistical power to detect changes in some of the parameters. Therefore, we performed a *post hoc* analysis of pooled data from the phase II and III trials [29, 30] to further examine the efficacy and safety of zonisamide in DLB patients with parkinsonism using a larger sample size. Specifically, regarding efficacy, we aimed to determine whether zonisamide improves symptoms of parkinsonism, particularly, which motor symptoms improve with zonisamide, and whether zonisamide affects cognitive function. Regarding safety, we aimed to evaluate the differences in the incidences of overall and individual AEs between zonisamide and placebo.

## MATERIALS AND METHODS

### Data pooling for analysis

**Fig. 1:**
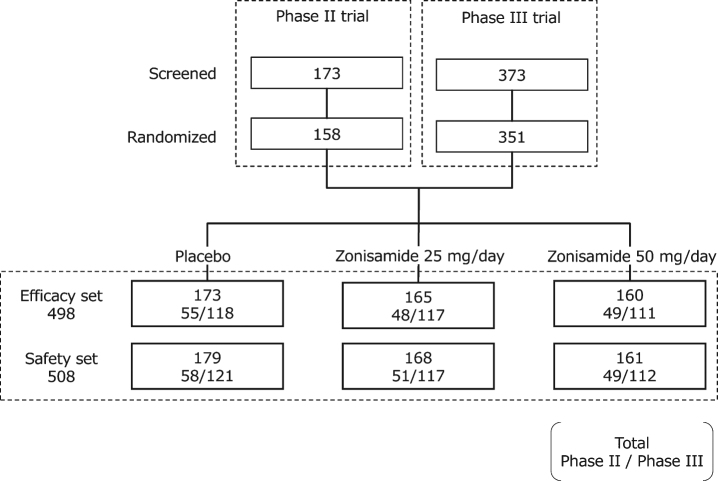
Patient disposition in the phase II and phase III trials used in the current analysis.

Data were pooled from the phase II (JapicCTI-122040) and phase III (JapicCTI-152839) trials of zonisamide in patients with DLB conducted in Japan [29, 30]; both trials were conducted in accord with the Declaration of Helsinki, Good Clinical Practice Ordinance and relevant regulations. Efficacy analyses were performed on the modified intention-to-treat populations from the two trials, which included all randomized patients who received at least one dose of the investigational product and had both baseline and at least one post-baseline UPDRS part III total scores (efficacy set). For the safety analysis, the safety populations from both trials were combined, which included randomized patients who received at least one dose of investigational product (safety set). The patient disposition for both trials is shown in Fig. 1.

### Patients and study design

Patients with probable DLB, diagnosed according to the Clinical Diagnostic Criteria for DLB (third report of the DLB consortium, 2005) [3], were eligible for inclusion in the primary trials. Both trials featured a 4-week run-in period followed by a 12-week treatment period with a double-blind, placebo-controlled, parallel-group, randomized, multicenter trial design. The key inclusion criteria were as follows: age 20–84 years (phase II trial) or 20–89 years (phase III trial); UPDRS part III total score ≥ 10; MMSE score 10–26 (phase II trial); and treatment with levodopa/decarboxylase inhibitor combination therapy for ≥ 12 weeks prior to screening, with the dose and regimen unchanged in the 2 weeks immediately prior to screening. In the phase III trial, patients with an MMSE score of < 10 were excluded. In the 4-week single-blind run-in period, all patients received placebo once daily to exclude a subsequent placebo effect. In the treatment period, patients were randomized 1:1:1 to zonisamide (25 or 50 mg) or placebo in a double-blind manner.

### Outcomes

The key outcome for the efficacy of zonisamide against parkinsonism in DLB in this analysis was the change from baseline at week 12 in UPDRS part III total score. To reduce inter-/intra-rater variability, training was conducted prior to the trial start. A more detailed analysis of the effects of zonisamide on the following four motor symptoms [31] was also performed: tremor (items 20, 21), rigidity (item 22), bradykinesia (items 23–26, 31), and postural instability/gait disturbance (PIGD) (items 29, 30). For each individual motor symptom, patients with zero or missing values throughout the trial were excluded from the analysis. Responders were determined based on *a*≥10%, ≥ 20%, or≥30% reduction from baseline in UPDRS part III total score at week 12, in reference to previous reports [16, 32] where missing values were imputed using the last observation carried forward (LOCF) method. Other efficacy outcomes were the change from baseline at week 12 (LOCF) in the Neuropsychiatric Inventory-10 (NPI-10) score [33], representing the behavioral and psychological symptoms of dementia (BPSD) rating scale; and in the MMSE score [34], representing cognitive function. Safety outcomes included the occurrence of AEs during the treatment period in each trial, coded according to the Medical Dictionary for Regulatory Activities, Japanese version, v19.1.

### Statistical analysis

All statistical analyses were performed using one-stage meta-analysis methods with individual patient data. Differences in patient demographics and clinical characteristics at baseline among the placebo and zonisamide 25 mg and 50 mg groups were analyzed using the Cochran–Mantel–Haenszel test stratified with trial for categorical variable and analysis of variance with trial as the fixed effect for continuous variables. Efficacy against parkinsonism was assessed using a mixed-effect model for repeated measures among the placebo and zonisamide 25 mg and 50 mg groups with treatment group, visit, trial, and treatment-by-visit interaction as fixed effects, and baseline value as a covariate. An unstructured covariance matrix was assumed, and the degree of freedom was estimated using Kenward–Roger’s approximation. *p*-values for the proportion of responders were calculated using logistic regression analysis with Firth’s penalized likelihood approach, with treatment group and trial as fixed effects and baseline value as a covariate. Statistical differences in MMSE and NPI-10 score changes between the zonisamide and placebo groups were evaluated by analysis of covariance with treatment group and trial as the fixed effects and baseline value as a covariate. Interaction *p*-values were calculated to evaluate the heterogeneity between the trials and were added to each original model. Statistical differences in the incidence of AEs between the zonisamide and placebo groups were analyzed using the Mantel–Haenszel test stratified by trial as a fixed effect. All statistical analyses were carried out at a two-sided significance level of 5%. No multiplicity adjustment for multiple treatment groups, outcomes, or visits was performed. All statistical analyses were performed using SAS version 9.4 (SAS Institute, Cary, NC, USA).

## RESULTS

### Patient demographics and baseline clinical characteristics

The efficacy set comprised 498 subjects (55.6% male) with a mean age of 76.6 years, mean DLB duration of 1.4 years, and mean levodopa dose of 262 mg/day (Table 1). The safety set comprised 508 subjects (Fig. 1). In the efficacy set, the mean baseline scores of the UPDRS part III total, MMSE, and NPI-10 were 31.5, 21.6, and 6.8, respectively. No differences in baseline parameters were observed among the three treatment groups (Table 1). At baseline, the number of subjects with existing tremor, particularly tremor at rest, was smaller than that observed for other symptoms or items (Table 2). In addition, the scores for “attention and calculation” and “drawing” in MMSE were relatively lower than those for other items (Table 2).

**Table 1:** Patient demographics and clinical characteristics at baseline

			Zonisamide		p^*^
	Total	Placebo	25 mg	50 mg
	(N = 498)	(N = 173)	(N = 165)	(N = 160)
Sex, male	277 (55.6)	99 (57.2)	97 (58.8)	81 (50.6)	0.290
Age, y
Mean	76.6±6.7	76.2±7.1	76.4±6.6	77.1±6.4	0.422
Range	55–89	56–89	55–89	59–88	—
≥65	472 (94.8)	161 (93.1)	157 (95.2)	154 (96.3)	0.427
≥75	320 (64.3)	108 (62.4)	105 (63.6)	107 (66.9)	0.688
Disease duration, y
DLB	1.4±1.7 (*n* = 497)	1.5±1.6	1.4±1.8	1.4±1.6 (*n* = 159)	0.943
Motor dysfunction	3.0±2.5 (*n* = 495)	3.0±2.7 (*n* = 171)	3.0±2.6 (*n* = 164)	3.0±2.3	0.993
Dementia	3.7±2.6 (*n* = 493)	3.7±2.6 (*n* = 172)	3.7±2.6 (*n* = 163)	3.6±2.6 (*n* = 158)	0.935
DLB core features
Fluctuating cognition	334 (67.1)	114 (65.9)	110 (66.7)	110 (68.8)	0.851
Visual hallucination	297 (59.6)	101 (58.4)	97 (58.8)	99 (61.9)	0.776
Parkinsonism	498 (100.0)	173 (100.0)	165 (100.0)	160 (100.0)	—
DLB suggestive features
Rapid eye movement sleep behavior disorder	235 (47.2)	87 (50.3)	80 (48.5)	68 (42.5)	0.336
Severe neuroleptic sensitivity	55 (11.0)	17 (9.8)	20 (12.1)	18 (11.3)	0.748
Concomitant drugs
Baseline levodopa dose, mg/day	262±153	257±154	256±159	273±147	0.510
Baseline levodopa-equivalent dose, mg/day	295±195	294±209	291±203	300±171	0.923
MAO-B inhibitor	20 (4.0)	7 (4.0)	7 (4.2)	6 (3.8)	0.966
Amantadine	25 (5.0)	6 (3.5)	10 (6.1)	9 (5.6)	0.497
Dopamine agonist	77 (15.5)	27 (15.6)	25 (15.2)	25 (15.6)	0.995
A2A receptor antagonist	8 (1.6)	5 (2.9)	2 (1.2)	1 (0.6)	0.213
Droxidopa	21 (4.2)	4 (2.3)	6 (3.6)	11 (6.9)	0.107
Anti-cholinergic drug	7 (1.4)	2 (1.2)	1 (0.6)	4 (2.5)	0.330
COMT inhibitor	24 (4.8)	13 (7.5)	5 (3.0)	6 (3.8)	0.120
Anti-dementia drug	361 (72.5)	122 (70.5)	125 (75.8)	114 (71.3)	0.500
Donepezil	260 (52.2)	82 (47.4)	85 (51.5)	93 (58.1)	0.136
Memantine	54 (10.8)	20 (11.6)	20 (12.1)	14 (8.8)	0.581
Rivastigmine	48 (9.6)	18 (10.4)	21 (12.7)	9 (5.6)	0.090
Galantamine	37 (7.4)	15 (8.7)	13 (7.9)	9 (5.6)	0.547
Yokukansan^†^	95 (19.1)	35 (20.2)	31 (18.8)	29 (18.1)	0.891
Other central nervous system drugs	207 (41.6)	68 (39.3)	74 (44.8)	65 (40.6)	0.579
Scores at baseline
UPDRS part III total	31.5±11.6	30.8±10.6	32.3±12.5	31.6±11.8	0.471
MMSE	21.6±5.2	22.1±5.2	21.0±5.7	21.8±4.7	0.180
NPI-10	6.8±8.7	6.7±8.3	7.2±10.2	6.5±7.5	0.743

Data are reported as mean±standard deviation or n (%). ^*^Analysis of variance for continuous variables and Cochran–Mantel–Haenszel test for categorical variables. ^†^Traditional Japanese herbal medicine. A2A, adenosine A_2A_; COMT, catechol-O-methyltransferase; DLB, dementia with Lewy bodies; MAO-B, monoamine oxidase B; MMSE, Mini-Mental State Examination; NPI, Neuropsychiatric Inventory; UPDRS, Unified Parkinson’s Disease Rating Scale.

**Table 2:** Baseline scores for individual assessment items

			Zonisamide
	Placebo		25 mg		50 mg
	(N = 173)		(N = 165)		(N = 160)
	Baseline score (mean±SD)	(n)	Baseline score (mean±SD)	(n)	Baseline score (mean±SD)	(n)
UPDRS part III
18. Speech	1.29±0.69	(152)	1.35±0.70	(146)	1.38±0.62	(145)
19. Facial expression	1.66±0.77	(166)	1.89±0.88	(159)	1.70±0.80	(157)
20. Tremor at rest	2.33±2.60	(96)	2.30±2.35	(87)	2.29±2.40	(86)
21. Action or postural tremor	1.70±1.08	(138)	1.79±1.21	(128)	1.83±1.21	(122)
22. Rigidity	7.16±3.14	(171)	7.40±3.55	(159)	7.08±3.42	(155)
23. Finger taps	3.14±1.69	(166)	3.30±1.61	(160)	3.08±1.54	(159)
24. Hand movement	2.67±1.47	(165)	2.98±1.61	(158)	2.81±1.42	(152)
25. Rapid alternating movements of hands (pronate/supinate)	3.23±1.61	(171)	3.44±1.50	(161)	3.34±1.67	(158)
26. Leg agility	2.77±1.44	(168)	2.89±1.69	(159)	2.84±1.36	(150)
27. Arising from chair	1.24±0.97	(124)	1.47±1.06	(116)	1.37±1.01	(121)
28. Posture	1.43±0.65	(166)	1.57±0.72	(161)	1.55±0.83	(158)
29. Gait	1.43±0.67	(165)	1.46±0.77	(158)	1.50±0.75	(158)
30. Postural stability	1.58±0.81	(158)	1.58±0.94	(150)	1.53±0.86	(148)
31. Body bradykinesia/hypokinesia	1.83±0.77	(171)	1.98±0.90	(164)	2.01±0.78	(159)
Individual motor symptom
Tremor	3.04±2.80	(151)	3.11±2.89	(138)	3.11±2.90	(135)
Rigidity	7.16±3.14	(171)	7.40±3.55	(159)	7.08±3.42	(155)
Bradykinesia	13.25±5.78	(173)	14.16±6.21	(165)	13.69±5.65	(160)
PIGD	2.84±1.38	(171)	2.87±1.63	(163)	2.91±1.46	(159)
MMSE
1. Orientation to time	3.49±1.53	(168)	3.41±1.71	(157)	3.50±1.54	(155)
2. Orientation to place	4.19±1.06	(168)	3.83±1.29	(157)	3.93±1.18	(155)
3. Registration	2.89±0.38	(168)	2.80±0.53	(157)	2.87±0.46	(155)
4. Attention and	2.08±1.74	(168)	1.94±1.65	(157)	1.93±1.64	(155)
calculation
5. Recall	1.80±1.07	(168)	1.68±1.17	(157)	1.66±1.10	(155)
6. Naming	1.99±0.08	(168)	1.98±0.19	(157)	2.00±0.00	(155)
7. Repetition	0.99±0.11	(168)	0.98±0.14	(157)	0.98±0.14	(155)
8. Comprehension	2.45±0.86	(168)	2.48±0.72	(157)	2.52±0.68	(155)
9. Reading	0.96±0.20	(168)	0.92±0.27	(157)	0.97±0.16	(155)
10. Writing	0.74±0.44	(168)	0.69±0.46	(157)	0.77±0.42	(155)
11. Drawing	0.61±0.49	(168)	0.57±0.50	(157)	0.68±0.47	(155)
NPI-10
A. Delusions	0.51±1.52	(166)	0.48±1.38	(158)	0.37±1.17	(156)
B. Hallucinations	1.01±1.77	(166)	1.03±1.93	(158)	0.85±1.63	(156)
C. Agitation/aggression	0.49±1.41	(166)	0.62±1.67	(158)	0.46±1.20	(156)
D. Depression/dysphoria	0.80±1.86	(166)	0.68±1.69	(158)	0.75±1.39	(156)
E. Anxiety	0.93±1.90	(166)	0.76±1.73	(158)	0.82±2.07	(156)
F. Elation/euphoria	0.08±0.40	(166)	0.13±0.86	(158)	0.02±0.24	(156)
G. Apathy/indifference	1.78±2.81	(166)	1.89±3.02	(158)	2.32±3.19	(156)
H. Disinhibition	0.06±0.29	(166)	0.19±1.00	(158)	0.04±0.22	(156)
I. Irritability/lability	0.41±1.34	(166)	0.37±1.35	(158)	0.29±1.14	(156)
J. Aberrant motor behavior	0.52±1.64	(166)	0.67±2.05	(158)	0.65±2.06	(156)

For each UPDRS item and individual motor symptom, patients with zero or missing values throughout the trial were excluded from the analysis. MMSE, Mini-Mental State Examination; NPI, Neuropsychiatric Inventory; PIGD, postural instability/gait disturbance; SD, standard deviation; UPDRS, Unified Parkinson’s Disease Rating Scale.

### Effect on parkinsonism

**Fig. 2:**
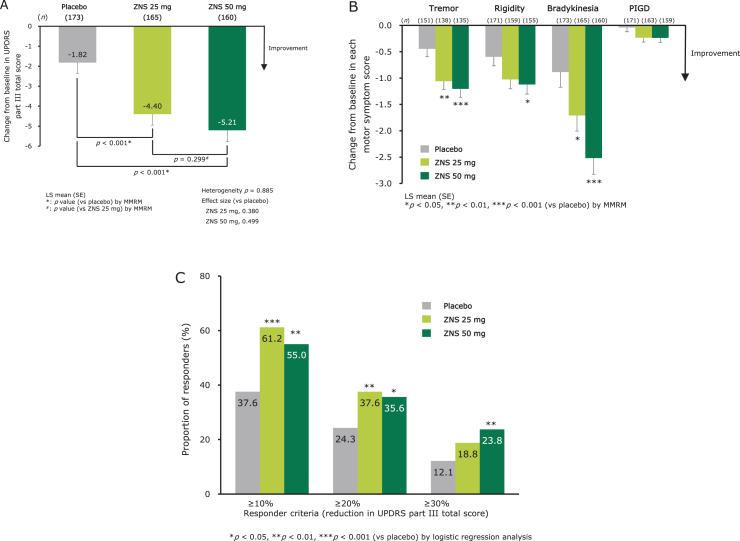
Efficacy of zonisamide for parkinsonism as measured by change from baseline at week 12 in UPDRS part III total score (A), individual motor symptom scores (B), and proportion of responders at week 12 (LOCF) (C). LS means (SE) and *p*-values for change from baseline in UPDRS scores were calculated using an MMRM with treatment group, visit, trial, and treatment-by-visit interaction as fixed effects, and baseline score as a covariate. An unstructured covariance matrix was assumed and the degree of freedom was estimated using Kenward–Roger’s approximation. Heterogeneity between the trials was evaluated using *p*-value for trial-by-treatment-by-visit interaction, which was added to the original MMRM model. *p*-values for the proportion of responders were calculated using logistic regression analysis with Firth’s penalized likelihood approach, with treatment group and trial as fixed effects and baseline score as a covariate. Responder: UPDRS part III total score change from baseline ≥ 10%, ≥ 20%, or ≥ 30%. LS mean, least-squares mean; LOCF, last observation carried forward; MMRM, mixed-effect model for repeated measures; PIGD, postural instability/gait disturbance; SE, standard error; UPDRS, Unified Parkinson’s Disease Rating Scale; ZNS, zonisamide.

Zonisamide significantly decreased the UPDRS part III total score compared with placebo, with no significant difference in score change observed between the zonisamide 25 mg and 50 mg groups (Fig. 2A). Zonisamide significantly improved individual motor symptom scores at week 12 for tremor (25 mg and 50 mg), rigidity (50 mg), and bradykinesia (25 mg and 50 mg) compared with placebo. No significant change in PIGD was observed in either zonisamide group (Fig. 2B). The score changes from baseline at week 12 in UPDRS part III items are shown in Supplementary Figure 1A.

The responder proportions (defined as a reduction in UPDRS part III total score ≥ 10% from baseline) at week 12 (LOCF) were 61.2% (*p* < 0.001 versus placebo) for zonisamide 25 mg, 55.0% (*p* = 0.002) for zonisamide 50 mg, and 37.6% for placebo (Fig. 2C), indicating a significant effect for both doses of zonisamide compared with placebo. In responders, the score changes in UPDRS part III total score from baseline at week 12 (LOCF) were –8.0 (0.5) (least squares mean [standard error]), –9.4 (0.5), and –8.3 (0.6) for zonisamide 25 mg, zonisamide 50 mg, and placebo, respectively. The responder proportions by other responder criteria (defined as a reduction in UPDRS part III total score≥20% or≥30% from baseline) are shown in Fig. 2C.

### Effect on cognitive function

**Fig. 3:**
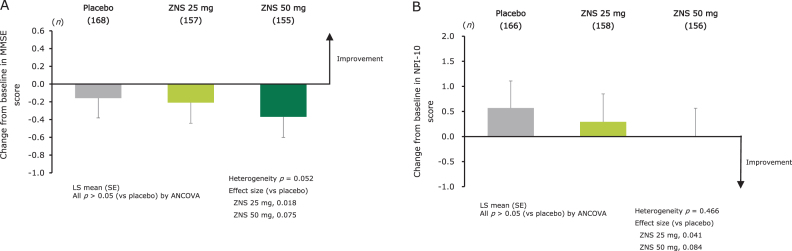
Effect of zonisamide on cognitive function as measured by change from baseline at week 12 in MMSE total score (A), and on BPSD as measured by change from baseline at week 12 in NPI-10 total score (B). LS means (SE) and *p*-values for change from baseline were calculated using an ANCOVA model with treatment group and trial as fixed effects, and baseline score as covariate. Heterogeneity between the trials was evaluated using *p*-value for trial-by-treatment interaction, which was added to the original ANCOVA model. ANCOVA, analysis of covariance; BPSD, behavioral and psychological symptoms of dementia; LS mean, least-squares mean; MMSE, Mini-Mental State Examination; NPI, Neuropsychiatric Inventory; SE, standard error; ZNS, zonisamide.

No significant difference in the change from baseline at week 12 in MMSE score was observed between the zonisamide groups and placebo (Fig. 3A). The score changes from baseline at week 12 in the MMSE individual items are shown in Supplementary Figure 1B.

### Effect on BPSD

No significant difference in the change from baseline at week 12 in NPI-10 score was observed between the zonisamide and placebo groups (Fig. 3B). The score changes from baseline at week 12 in the NPI-10 individual items are shown in Supplementary Figure 1C.

### Safety

Overall AEs are shown in Table 3. The incidence of AEs was 48.0%, 47.0%, and 57.8% in the placebo, zonisamide 25 mg, and 50 mg groups, respectively, with no significant differences observed between the zonisamide and placebo groups. With respect to the in-cidence of common AEs reported among the three treatment groups, decreased appetite was shown to have an increased frequency in the zonisamide 50 mg group compared with placebo, but for other AEs, no differences were observed between zonisamide and placebo (Table 3). The incidence of AEs classified as neurologic or psychiatric is shown in Table 4.

**Table 3:** Incidence of adverse events (overall and common adverse events)

						Zonisamide
	Placebo		25 mg				50 mg					Total
	(*N* = 179)		(*N* = 168)				(*N* = 161)					(*N* = 329)
	*n* (%)	*n* (%)	RR	[95% CI]	*p*	*n* (%)	RR	[95% CI]	*p*	*n* (%)	RR	[95% CI]	*p*
Any AEs	86 (48.0)	79 (47.0)	1.0	[0.8–1.2]	0.846	93 (57.8)	1.2	[1.0–1.5]	0.069	172 (52.3)	1.1	[0.9–1.3]	0.362
Common AEs^*^
Nasopharyngitis	11 (6.2)	12 (7.1)	1.2	[0.5–2.6]	0.695	16 (9.9)	1.6	[0.8–3.4]	0.200	28 (8.5)	1.4	[0.7–2.7]	0.336
Appetite decreased	2 (1.1)	6 (3.6)	3.2	[0.7–15.9]	0.149	11 (6.8)	6.2	[1.4–27.6]	0.018	17 (5.2)	4.6	[1.1–20.0]	0.039
Contusion	10 (5.6)	9 (5.4)	1.0	[0.4–2.3]	0.943	3 (1.9)	0.3	[0.1–1.2]	0.095	12 (3.7)	0.7	[0.3–1.5]	0.318
Weight decreased	0 (0.0)	3 (1.8)	4.3	[0.5–38.4]	0.191	6 (3.7)	7.9	[1.0–63.3]	0.050	9 (2.7)	5.6	[0.7–43.1]	0.099
Fall	4 (2.2)	3 (1.8)	0.8^†^	[0.2–3.3]^†^	0.789^†^	5 (3.1)	1.3	[0.4–4.2]	0.644	8 (2.4)	1.0	[0.3–2.8]	0.953
Somnolence	3 (1.7)	6 (3.6)	2.2	[0.5–8.6]	0.279	2 (1.2)	0.8	[0.1–4.5]	0.762	8 (2.4)	1.5	[0.4–5.5]	0.574
Diarrhea	1 (0.6)	3 (1.8)	2.1	[0.4–11.4]	0.378	3 (1.9)	2.2	[0.4–11.9]	0.352	6 (1.8)	1.9	[0.4–9.1]	0.414
Back pain	2 (1.1)	4 (2.4)	1.8	[0.4–7.2]	0.432	2 (1.2)	1.1	[0.2–5.4]	0.896	6 (1.8)	1.3	[0.3–4.8]	0.723
Pneumonia	1 (0.6)	3 (1.8)	2.1	[0.4–11.4]	0.378	2 (1.2)	1.9^†^	[0.2–13.9]^†^	0.549^†^	5 (1.5)	1.6	[0.3–8.0]	0.547
Dental caries	2 (1.1)	5 (3.0)	2.1	[0.5–8.3]	0.279	0 (0.0)	0.2^†^	[0.0–4.6]^†^	0.330^†^	5 (1.5)	1.1	[0.3–4.3]	0.896

For cells with zero frequency, the value 0.5 was added (Woolf continuity correction). Adjusted RR (95% CI) and *p*-values versus placebo group were calculated using the Mantel–Haenszel method, stratified by trial as a fixed effect. ^*^Ten most common AEs in the zonisamide total group. ^†^Unadjusted RR (95% CI) and *p*-values were calculated using Wald’s method if the RR was not calculated in either of the trials. AE, adverse event; CI; confidence interval; RR, risk ratio.

**Table 4:** Common neurologic and psychiatric adverse events

Common neurologic and psychiatric AEs^*^	Zonisamide
	Placebo	25 mg	50 mg	Total
	(N = 179)	(N = 168)	(N = 161)	(N = 329)
	*n* (%)	*n* (%)	*n* (%)	*n* (%)
Somnolence	3 (1.7)	6 (3.6)	2 (1.2)	8 (2.4)
Hallucination	1 (0.6)	1 (0.6)	2 (1.2)	3 (0.9)
Psychiatric symptom^†^	2 (1.1)	2 (1.2)	1 (0.6)	3 (0.9)
Insomnia	1 (0.6)	2 (1.2)	1 (0.6)	3 (0.9)
Dizziness	1 (0.6)	2 (1.2)	0 (0)	2 (0.6)
Dysgeusia	0 (0)	2 (1.2)	0 (0)	2 (0.6)
Delusion	2 (1.1)	0 (0)	0 (0)	0 (0)

^*^In≥1% of patients in either group. ^†^Aggravation of existing symptoms. AE, adverse event.

## DISCUSSION

In the present analysis, the characteristics of the patient population showed similarities to those in previous studies on DLB patients [6, 35, 36], such as a relatively low prevalence of tremor (particularly tremor at rest) and relatively low MMSE item scores of “attention and calculation” and “drawing” (indicating deficits in attention, executive function, and visuoperceptual ability). These characteristics are considered to represent the typical symptomatic features of DLB. However, in this analysis, a lower NPI-10 score was observed compared with previous studies on DLB patients [7, 36]. The difference may be associated with the inclusion criteria “UPDRS part III total score ≥ 10” and primary endpoint “the change from baseline at week 12 in UPDRS part III total score” in the primary trials.

Regarding whether the zonisamide 25 and 50 mg groups had improved symptoms of parkinsonism, and which motor symptoms were improved, in our analysis, zonisamide in both groups was shown to improve symptoms of parkinsonism, particularly bradykinesia and tremor (motor symptoms), with small differences in efficacy on parkinsonism observed between the 25 mg and 50 mg groups. Furthermore, the proportion of responders and changes in score among responders (≥ 10% reduction in UPDRS part III total score) who received zonisamide were comparable to those reported in a previous study of dopaminergic treatment in patients with DLB who received levodopa [15, 16]. Additionally, subgroup analysis among populations by severity of cognitive impairment or BPSD and selected baseline factors showed a reduction in UPDRS part III total scores in many of the patient populations receiving zonisamide (Supplementary Figures 2, 3). Based on these results, further studies are expected to clarify the details of zonisamide efficacy. Regarding the effect of zonisamide on parkinsonism in patients with DLB, the proportion of responders with≥30% reduction in UPDRS part III total score was lower than that reported in PD patients from previous studies [17, 18], and similar results were observed in the levodopa study [15].

With respect to the effect of zonisamide on cognitive function, no significant difference was observed between zonisamide (either dose) and placebo on the change in MMSE score. This finding differed from the outcome observed in the phase III trial, where zonisamide 50 mg significantly decreased the MMSE score compared with placebo [30]. Furthermore, in the analysis of the MMSE items and the subgroup analysis among patients with severe cognitive impairment, no differences in MMSE score change were observed between the zonisamide 25 mg or 50 mg groups and the placebo group (Supplementary Figures 1, 2). These findings, alongside the low frequency of cognitive AEs, indicate that zonisamide does not worsen cognitive function (and also does not improve cognitive impairment).

Regarding the effect of zonisamide on BPSD, no difference in NPI-10 score was observed for either dose of zonisamide versus placebo. Furthermore, in the analysis of the NPI-10 items and the subgroup analysis among patients with severe BPSD, no differences in NPI-10 score change were observed between the zonisamide 25 mg or 50 mg groups and the placebo group (Supplementary Figures 1, 2). Taken together with the low frequency of psychiatric AEs reported in the two trials, it appears that zonisamide does not worsen BPSD in patients with DLB (and also does not improve BPSD), in contrast to a previous study, which reported an association between levodopa and increased risk of psychiatric AEs in DLB patients [16].

A low incidence of each AE was observed among patients treated with zonisamide in the present study. With the exception of decreased appetite in patients receiving zonisamide 50 mg, the incidence of AEs did not differ between either zonisamide group and pla-cebo. In addition to the low frequency of AEs related to cognitive function and BPSD discussed above, the incidence of other AEs related to characteristic symptoms of DLB, such as autonomic dysfunction [2], was also low, supporting the safety of zonisamide in the current study population.

When evaluating the differences between the two doses of zonisamide, our findings show that the cha-nge in UPDRS part III total score was greater in the 50 mg group versus the 25 mg group; however, the difference was not statistically significant, suggesting no difference in efficacy. Regarding safety, although there was no significant difference in the overall incidence rate of adverse events, that in the 50 mg group was slightly higher than that in the 25 mg group, and the incidence rate of decreased appetite was significantly higher in the 50 mg group versus the placebo group. These findings suggest that the recommended dose of zonisamide for DLB patients with parkinsonism is 25 mg.

The present study had some limitations, including the *post hoc* study design and the inclusion of only Japanese patients, which limits the generalizability of the results. Furthermore, the trials enrolled participants from essentially the same patient population using the same inclusion/exclusion criteria, and the 12-week treatment periods were relatively short. Future studies with longer duration in a broader patient population are therefore required, and real-world study data may also contribute to determining the actual benefits of zonisamide to patients and caregivers.

In conclusion, the outcomes of this *post hoc* analysis indicate that zonisamide as adjunct therapy to levodopa is efficacious for the treatment of parkinsonism with DLB, particularly the management of bradykinesia, tremor, and rigidity. Importantly, exacerbation of psychiatric symptoms, which is typically associated with levodopa use, was not observed. Zonisamide was shown to be well tolerated in patients with DLB with parkinsonism, with no new safety signals identified in the present analysis. Authors’ disclosures available online (https://www.j-alz.com/manuscript-disclosures/20-0893r1).

## Supplementary Material

Supplementary Material
