# Supplementary material for: Efficacy and Safety of Zonisamide in Dementia with Lewy Bodies Patients with Parkinsonism: A Post Hoc Analysis of Two Randomized, Double-Blind, Placebo-Controlled Trials
Source: J Alzheimers Dis. 2021 Jan 19;79(2):627–37. doi: 10.3233/JAD-200893 (PMC7902955; doi:10.3233/JAD-200893)
Supplement: Supplementary Material [file jad-79-jad200893-s001.pdf]

Supplementary Material

Efficacy and Safety of Zonisamide in Dementia with Lewy Bodies Patients with Parkinsonism: A Post Hoc Analysis of Two Randomized, Double-Blind, Placebo-Controlled Trials

Supplementary Figure 1. Change from baseline in individual item scores for (A) UPDRS part III, (B) MMSE, and (C) NPI-10 at week 12. For each UPDRS item, patients with all zero or missing values throughout the study were excluded from the analysis. ANCOVA, analysis of covariance; LS mean; least-squares mean; MMSE, Mini-Mental State Examination; MMRM, mixed-effect model for repeated measures; NPI, Neuropsychiatric Inventory; SE, standard error; UPDRS, Unified Parkinson's Disease Rating Scale; ZNS, zonisamide.

A

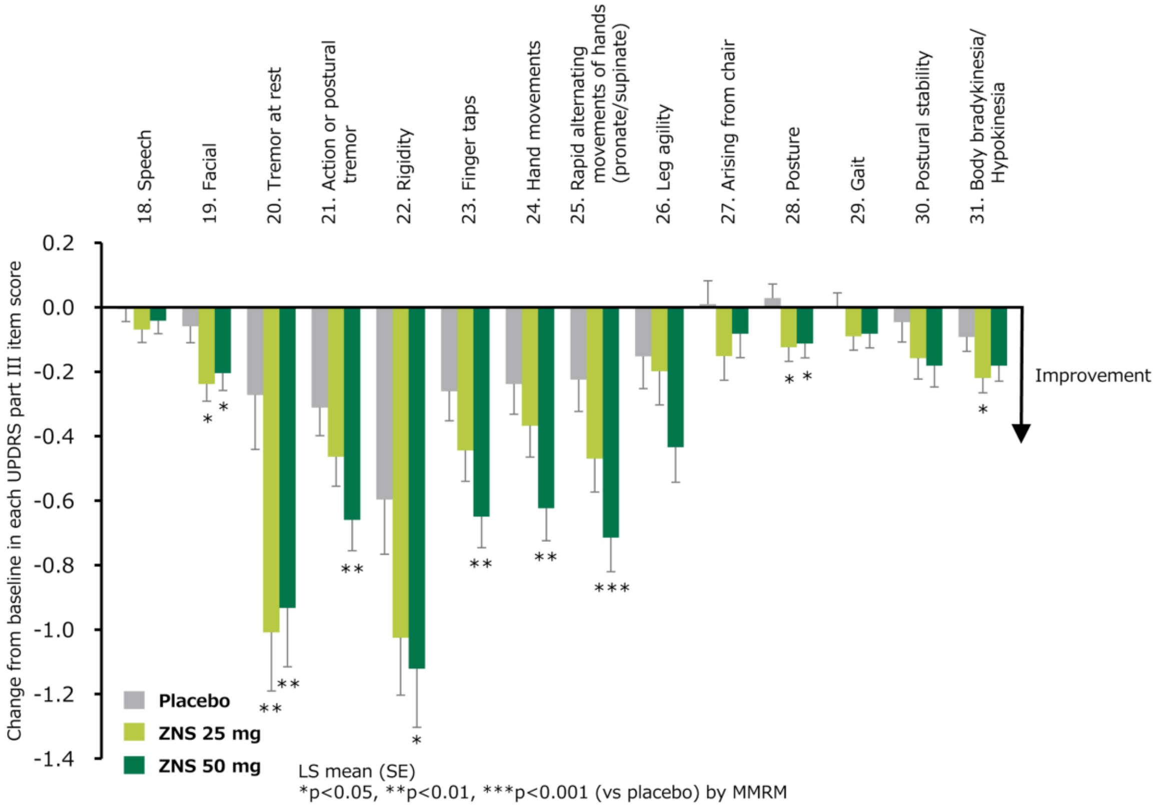

B

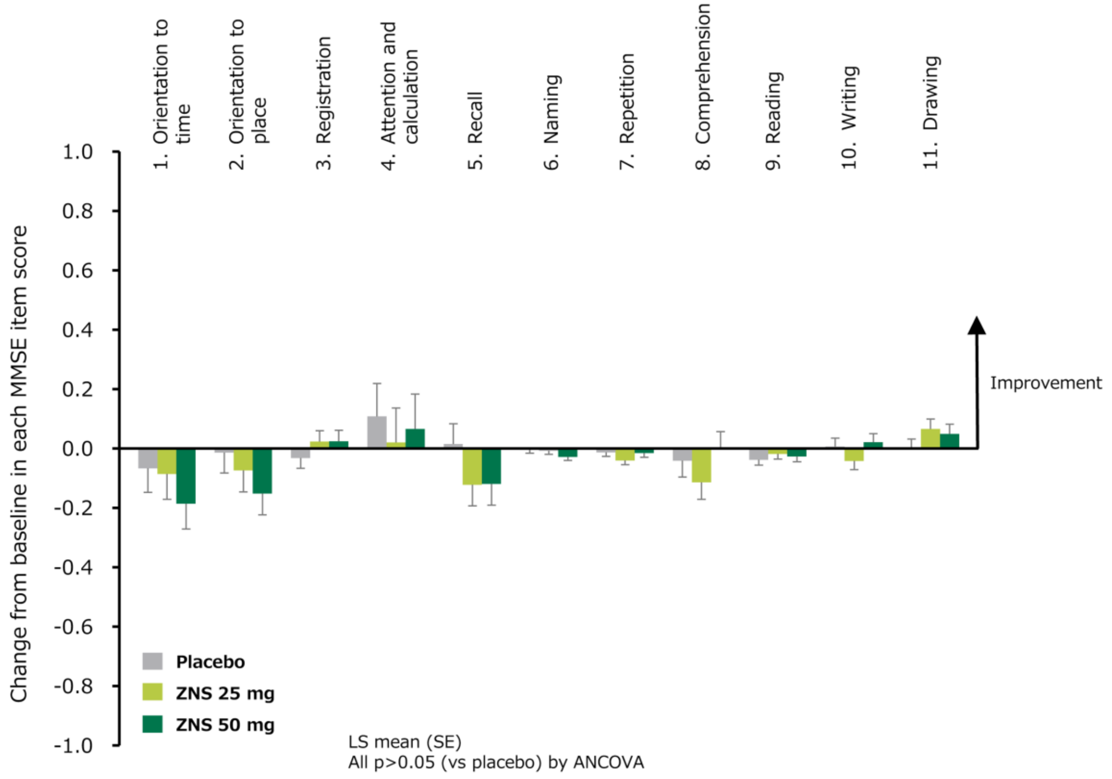
1. Orientation to time
2. Orientation to place
3. Registration
4. Attention and calculation
5. Recall
6. Naming
7. Repetition
8. Comprehension
9. Reading
10. Writing
11. Drawing
1.0
0.8
0.6
0.4
0.2
0.0
-0.2
-0.4
-0.6
-0.8
-1.0
Change from baseline in each MMSE item score
Improvement
Placebo
ZNS 25 mg
ZNS 50 mg
LS mean (SE)
All p>0.05 (vs placebo) by ANCOVA

C

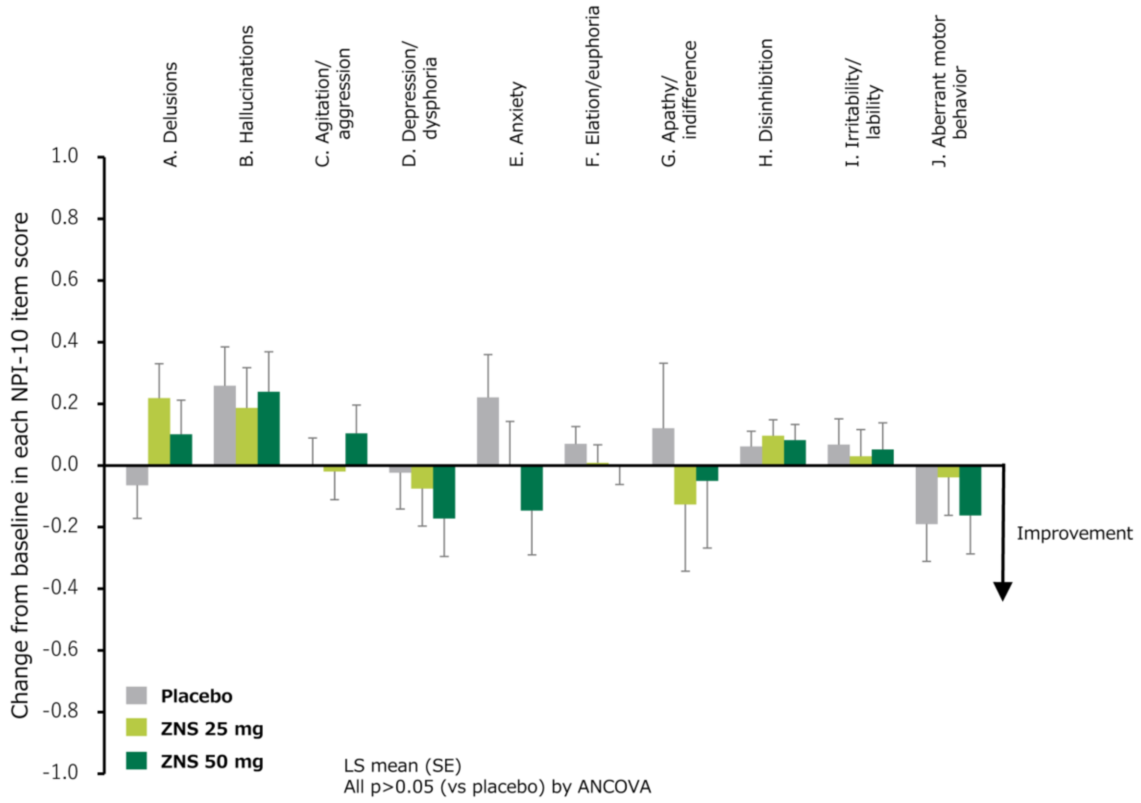
A. Delusions
B. Hallucinations
C. Agitation/ aggression
D. Depression/ dysphoria
E. Anxiety
F. Elation/euphoria
G. Apathy/ indifference
H. Disinhibition
I. Irritability/ lability
J. Aberrant motor behavior
1.0
0.8
0.6
0.4
0.2
0.0
-0.2
-0.4
-0.6
-0.8
-1.0
Change from baseline in each NPI-10 item score
Improvement
Placebo
ZNS 25 mg
ZNS 50 mg
LS mean (SE)
All p>0.05 (vs placebo) by ANCOVA

Supplementary Figure 2. Subgroup analysis by severity of cognitive impairment and BPSD. Changes from baseline at week 12 are shown for (A) UPDRS part III total score by severity of cognitive impairment, (B) UPDRS part III total score by severity of BPSD, (C) MMSE total score by severity of cognitive impairment, and (D) NPI-10 total score by severity of BPSD. ANCOVA, analysis of covariance; BPSD, behavioral and psychological symptoms of dementia; LS mean, least-squares mean; MMSE, Mini-Mental State Examination; MMRM, mixed-effect model for repeated measures; NPI, Neuropsychiatric Inventory; SE, standard error; UPDRS, Unified Parkinson's Disease Rating Scale; ZNS, zonisamide.

A

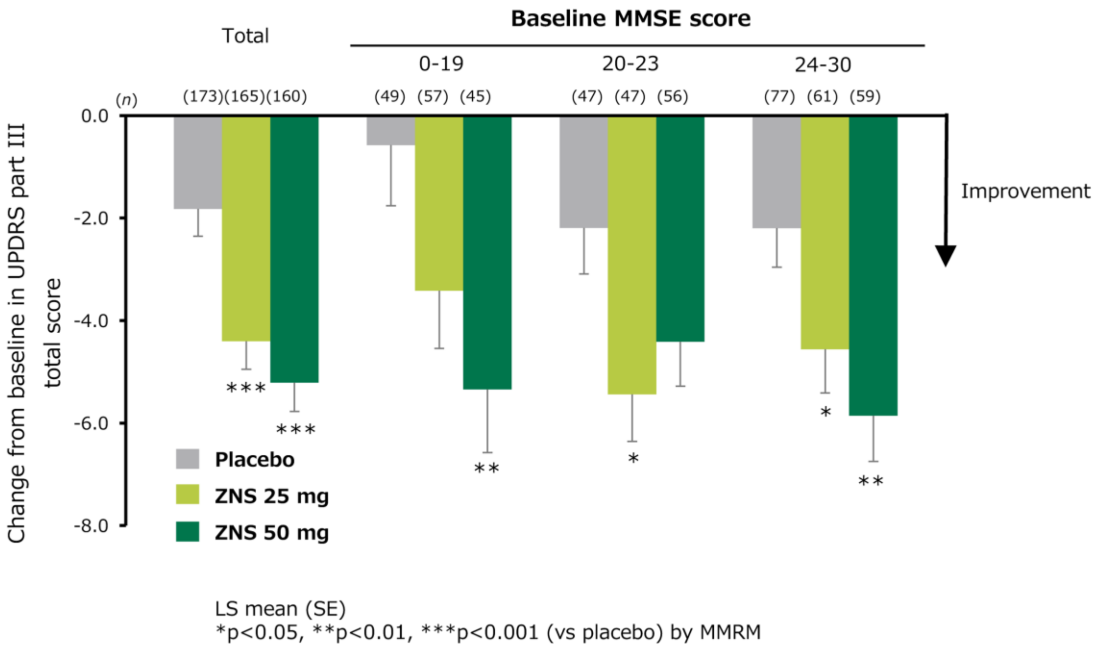

B

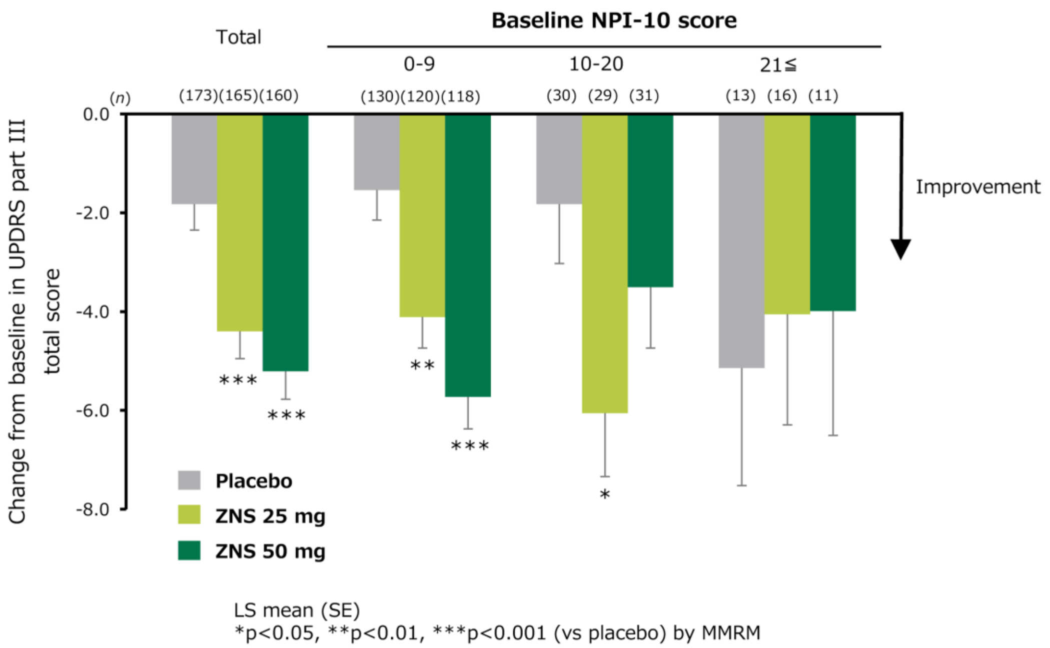

C

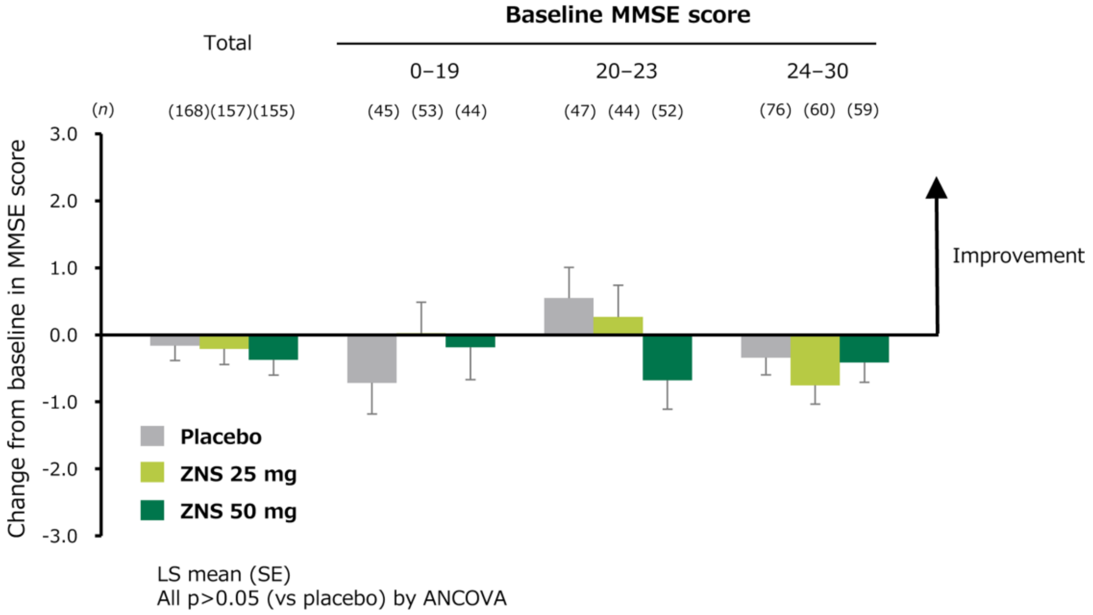
Baseline MMSE score
Total
0–19
20–23
24–30
(n)
(168)(157)(155)
(45) (53) (44)
(47) (44) (52)
(76) (60) (59)
Change from baseline in MMSE score
3.0
2.0
1.0
0.0
-1.0
-2.0
-3.0
Improvement
Placebo
ZNS 25 mg
ZNS 50 mg
LS mean (SE)
All p>0.05 (vs placebo) by ANCOVA

D

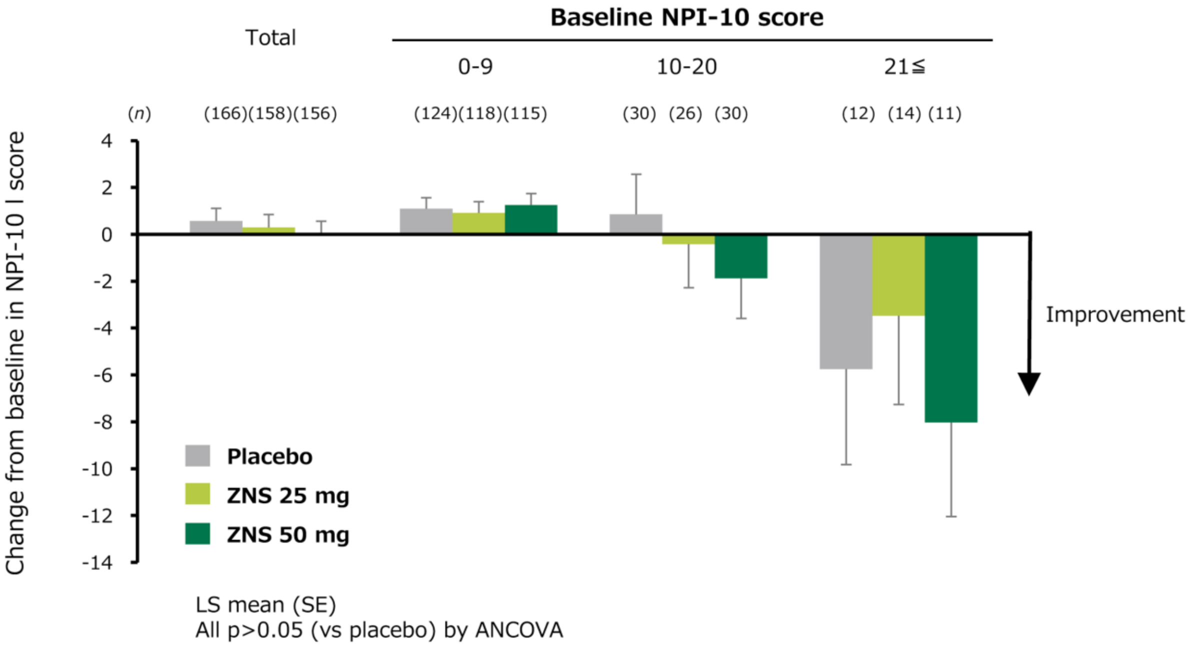
Baseline NPI-10 score
Total
0-9
10-20
21≦
(n)
(166)(158)(156)
(124)(118)(115)
(30) (26) (30)
(12) (14) (11)
Change from baseline in NPI-10 I score
4
2
0
-2
-4
-6
-8
-10
-12
-14
Improvement
Placebo
ZNS 25 mg
ZNS 50 mg
LS mean (SE)
All p>0.05 (vs placebo) by ANCOVA

Supplementary Figure 3. Subgroup analysis according to selected baseline factors. Changes from baseline at week 12 in UPDRS total score are shown stratified by (A) levodopa dose, (B) UPDRS part III total score, (C) presence of tremor, (D) age, (E) onset order of symptoms, and (F) concomitant use of antipsychotics. LS mean, least-squares mean; MMRM, mixed-effect model for repeated measures; SE, standard error; UPDRS, Unified Parkinson's Disease Rating Scale; ZNS, zonisamide.

A

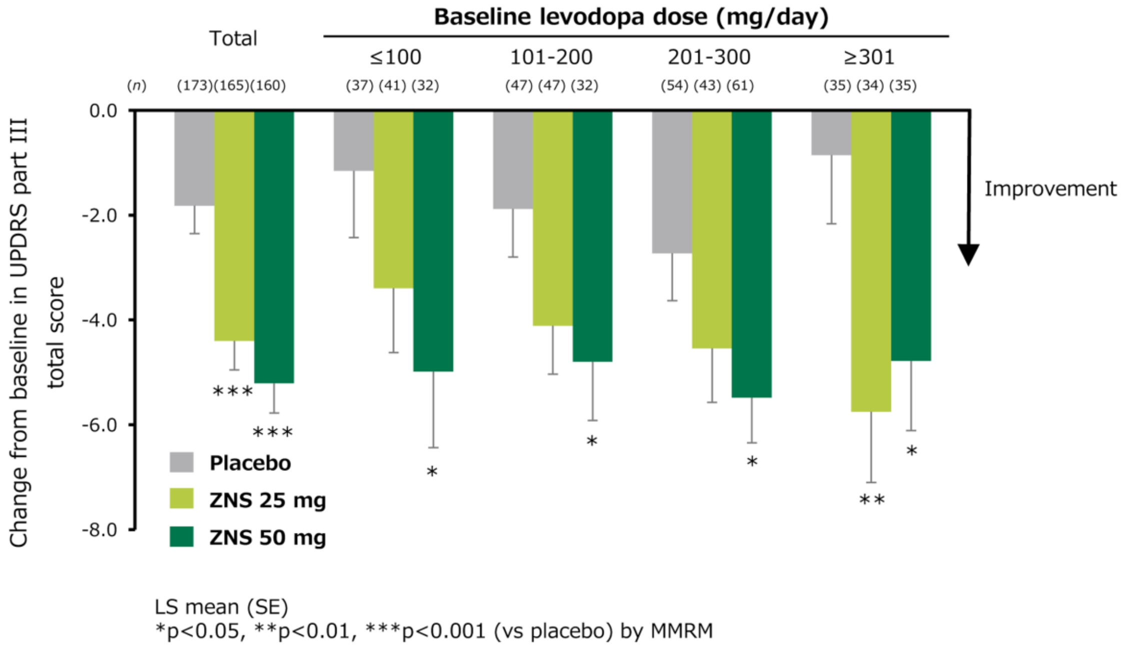

B

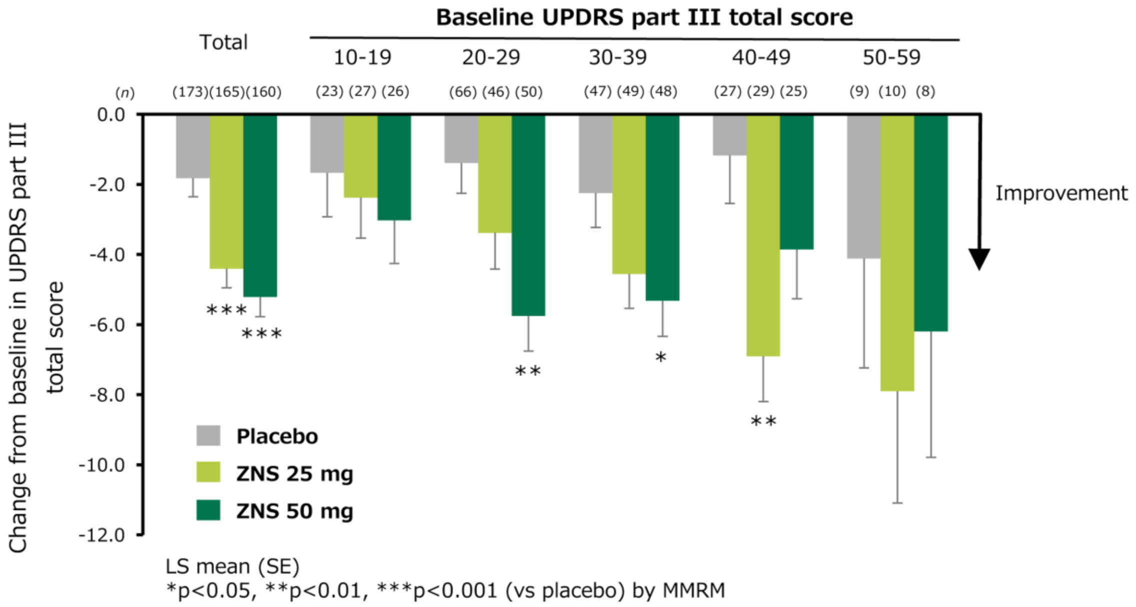

C

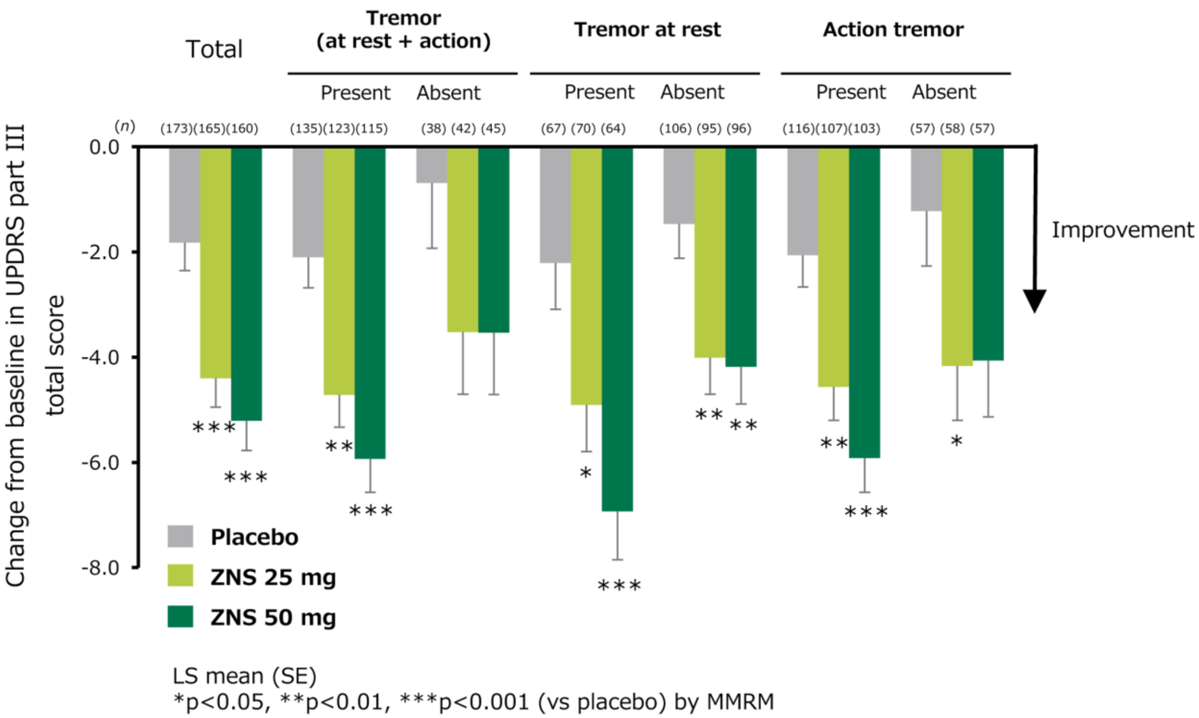
Total
Tremor (at rest + action)
Tremor at rest
Action tremor
Present
Absent
(n)
(173)(165)(160)
(135)(123)(115)
(38) (42) (45)
(67) (70) (64)
(106) (95) (96)
(116)(107)(103)
(57) (58) (57)
Change from baseline in UPDRS part III total score
0.0
-2.0
-4.0
-6.0
-8.0
Improvement
Placebo
ZNS 25 mg
ZNS 50 mg
LS mean (SE)
*p<0.05, **p<0.01, ***p<0.001 (vs placebo) by MMRM

D

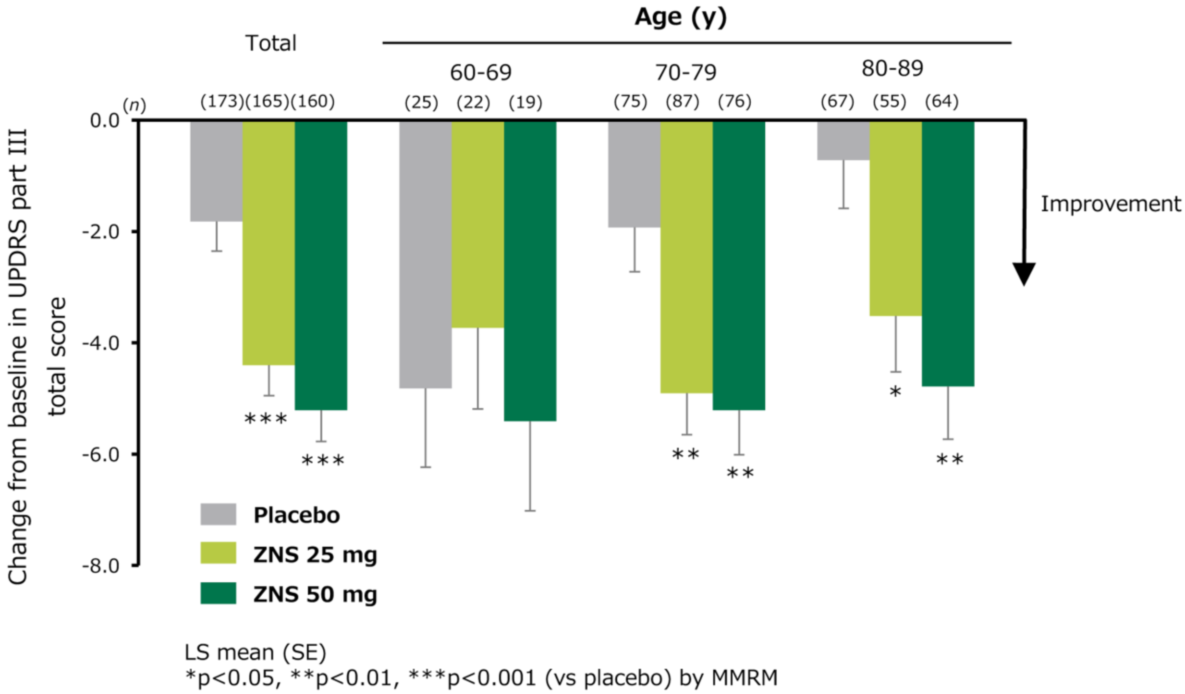
Total
Age (y)
60-69
70-79
80-89
(n)
(173)(165)(160)
(25) (22) (19)
(75) (87) (76)
(67) (55) (64)
Change from baseline in UPDRS part III total score
0.0
-2.0
-4.0
-6.0
-8.0
Improvement
Placebo
ZNS 25 mg
ZNS 50 mg
LS mean (SE)
*p<0.05, **p<0.01, ***p<0.001 (vs placebo) by MMRM

E

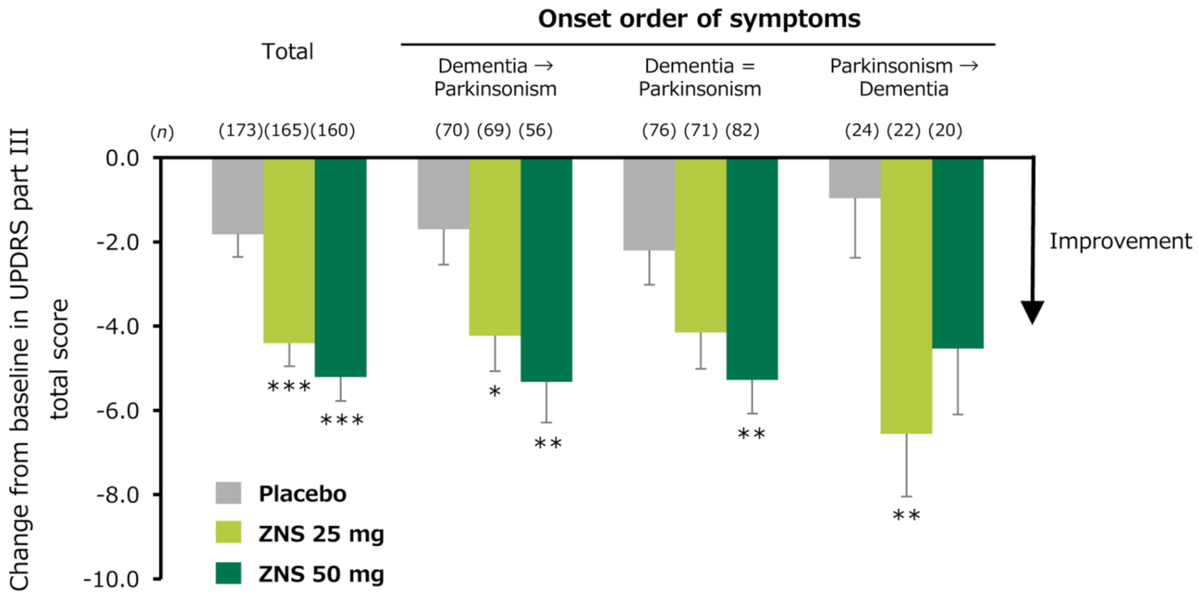

LS mean (SE)
*p<0.05, **p<0.01, ***p<0.001 (vs placebo) by MMRM

F

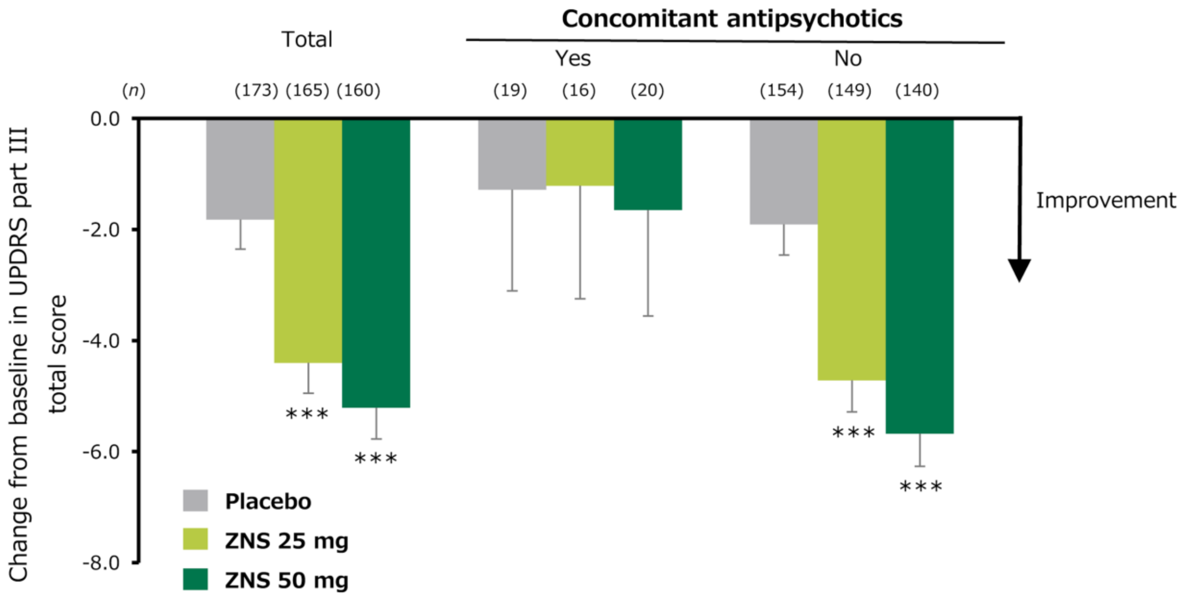

LS mean (SE)
***p<0.001 (vs placebo) by MMRM
